# Iterative rank-order normalization of gene expression microarray data

**DOI:** 10.1186/1471-2105-14-153

**Published:** 2013-05-07

**Authors:** Eric A Welsh, Steven A Eschrich, Anders E Berglund, David A Fenstermacher

**Affiliations:** 1 H. Lee Moffitt Cancer Center and Research Institute, University of South Florida, Tampa, FL, 33612, USA

**Keywords:** Microarray, Expression, Normalization, Affymetrix, GeneChip

## Abstract

**Background:**

Many gene expression normalization algorithms exist for Affymetrix GeneChip microarrays. The most popular of these is RMA, primarily due to the precision and low noise produced during the process. A significant strength of this and similar approaches is the use of the entire set of arrays during both normalization and model-based estimation of signal. However, this leads to differing estimates of expression based on the starting set of arrays, and estimates can change when a single, additional chip is added to the set. Additionally, outlier chips can impact the signals of other arrays, and can themselves be skewed by the majority of the population.

**Results:**

We developed an approach, termed IRON, which uses the best-performing techniques from each of several popular processing methods while retaining the ability to incrementally renormalize data without altering previously normalized expression. This combination of approaches results in a method that performs comparably to existing approaches on artificial benchmark datasets (i.e. spike-in) and demonstrates promising improvements in segregating true signals within biologically complex experiments.

**Conclusions:**

By combining approaches from existing normalization techniques, the IRON method offers several advantages. First, IRON normalization occurs pair-wise, thereby avoiding the need for all chips to be normalized together, which can be important for large data analyses. Secondly, the technique does not require similarity in signal distribution across chips for normalization, which can be important for maintaining biologically relevant differences in a heterogeneous background. Lastly, IRON introduces fewer post-processing artifacts, particularly in data whose behavior violates common assumptions. Thus, the IRON method provides a practical solution to common needs of expression analysis. A software implementation of IRON is available at [http://gene.moffitt.org/libaffy/].

## Background

An important first step of any microarray experiment is the normalization of the samples. Although the relative impacts differ from platform to platform and sample preparation, non-biological differences in microarray signals can stem from a variety of factors, such as: global constant background noise, non-specific binding signal, non-linear signal response between samples, bad spots on the chip due to dust or bubbles or rare manufacturing defects, labeling efficiency, hybridization efficiency, and RNA quality. While some can be difficult or impossible to detect and correct for computationally, most can be addressed to some extent by how the raw data is processed in order to yield the final transcript intensities. Thus, the methods used to post-process the raw data can have a large impact on the final biological signal. We also here want to make a clear distinction between what is commonly called “batch-effects” and the kind of variance that should be minimized with a good normalization method. Batch effects are as real as any biological signal, and are indistinguishable from biological signal without post-normalization interpretation of experiment-related metadata. As such, they are not suitable for removal by chip normalization methods. There are other tools which specifically focus on removing batch effect after initial chip post-processing, such as COMBAT [1], and our focus in this manuscript will be on removing non- batch-related technical variation.

**Figure 1:**
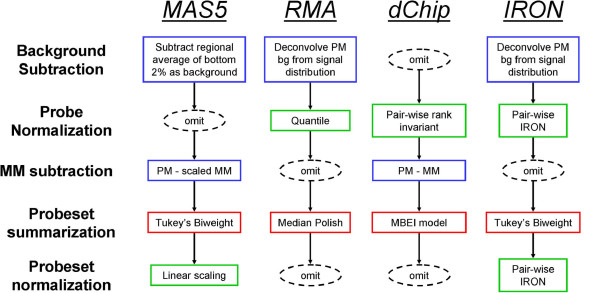
**Comparison of MAS5, RMA, dChip, and IRON microarray post-processing pipelines.** IRON combines components of both MAS5 and RMA, substituting a novel pair-wise iterative rank-order normalization method for normalization steps.

The three most commonly used software packages for processing Affymetrix microarrays, as evidenced by recently querying the GEO [2] and ArrayExpress [3] microarray repositories, are: RMA [4], MAS5 [5], and dChip [6]. Each of these employs different methods for background subtraction, signal normalization, and probeset summarization (an issue unique to Affymetrix arrays, where multiple probes for the same transcript are condensed into a single representative signal). A flowchart of these pipelines is given in Figure 1. There are various theoretical and empirical advantages and disadvantages to the various steps in each processing pipeline, many of which have been discussed previously [7,8], and will be further discussed below.

We present here a new pipeline, IRON (Iterative Rank-Order Normalization), which combines what we consider the most desirable steps of each pipeline, and further improves upon the normalization approach of dChip. We introduce a novel method for normalization of Affymetrix arrays based on rank-invariant probesets, combined with steps from both the RMA and MAS5 pipelines (Figure 1). Our design goals for IRON include (1) the ability to incrementally normalize additional data, (2) the ability to process as few as two chips without negatively impacting quality, (3) provide robust normalization under noisy or systematic effects as commonly seen in biologically complex datasets (e.g., samples with a complex or heterogeneous background), and (4) efficiently handle large numbers of samples. IRON seeks to avoid limitations of existing algorithms where possible, and selects the algorithm that best incorporates our design goals. Each step of the normalization and summarization algorithm is motivated by empirical examples and demonstrates the utility of the IRON approach.

This pipeline is implemented in the freely available libaffy C library and associated applications. A generic pipeline, lacking Affymetrix-specific modules, is also provided for use on non-Affymetrix datasets.

## Results and discussion

### RMA background subtraction does not introduce low-intensity variability

**Figure 2:**
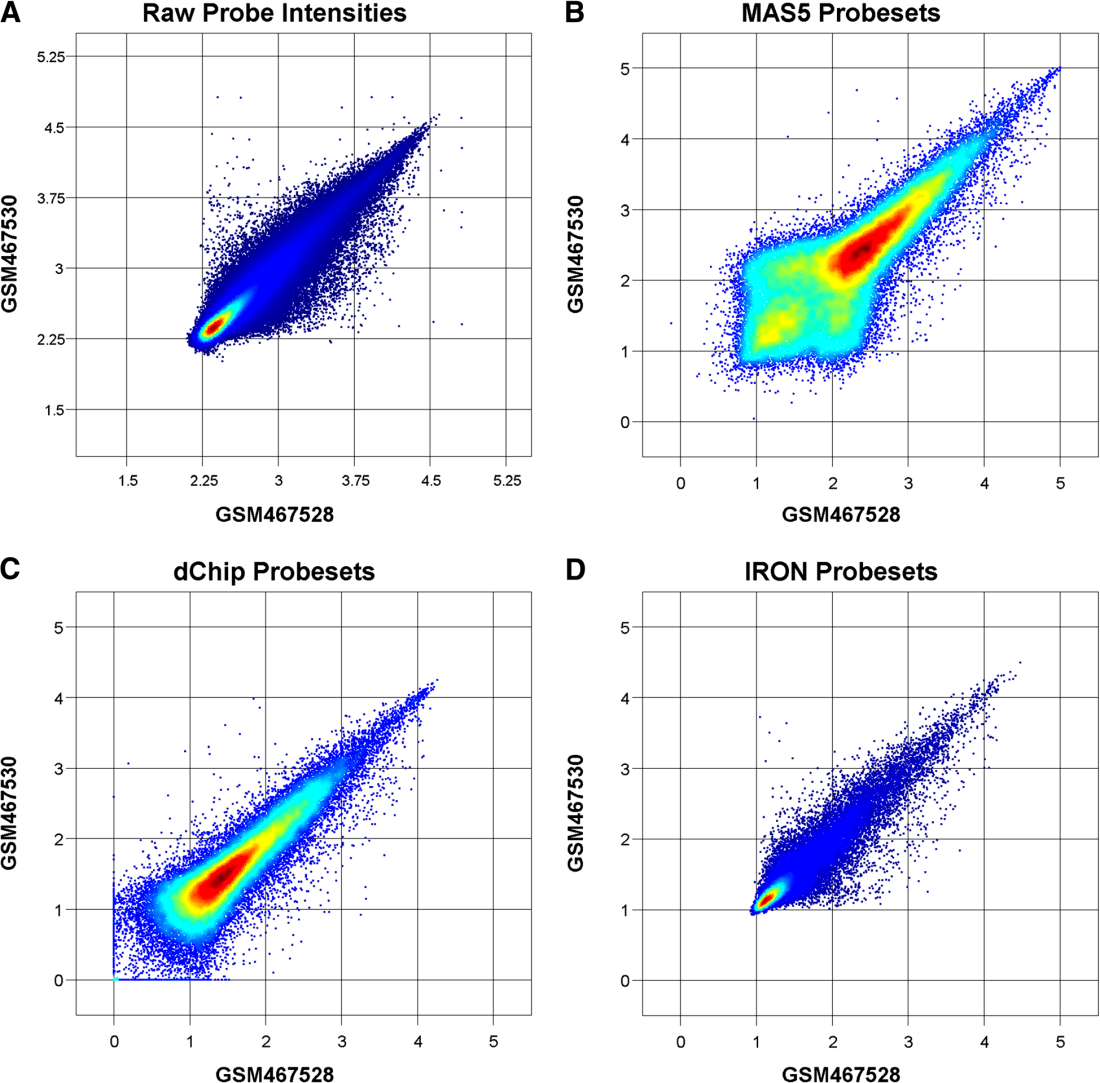
**Effect of background subtraction method on probeset distributions.** Color denotes spatial density (red: high, blue: low). Raw unprocessed log_10_ probe signal intensities are plotted in (**A**). MAS5 probesets (**B**) exhibit a markedly different distribution than the underlying probe-level data, with a large increase in variance at low intensities due to MM subtraction. dChip probesets (**C**) are similar to those of MAS5, despite the large difference in probeset summarization methodology, due to similar use of mis-match probe subtraction. IRON probesets (**D**), due to the use of RMA background subtraction, exhibit a distribution similar to that of the underlying probe-level data. RMA probesets (data not shown) produce a distribution highly similar to that of IRON, due to shared background subtraction methodology.

The choice of background subtraction methodology can have a large impact on the final processed signal intensities. For Affymetrix arrays, MM (mis-match) probes differ from PM (perfect match) probes by a single base in the center of the 25-mer. These probes were originally intended for use in estimating non-specific binding signal. However, the subtraction of MM intensities from PM intensities, exemplified by MAS5, has been shown to be less than optimal, due to the amount of target-gene specific binding present in the presumably non-specific MM signal [4]. Although Choe *et al*. [7] and Irizarry *et al*. [8] arrived at opposite conclusions regarding the suitability of MAS5 background subtraction, this can largely be explained by differences in the methodology used to determine differentially expressed genes. Both manuscripts agree that MAS5 background subtraction introduces high variability into low-intensity probes, which agrees with subjective visual inspection of resulting probeset scatterplots (Figure 2B).

RMA background subtraction deconvolves, in log_2_-space, a low-intensity normally distributed background from an exponentially decaying signal, ignoring the MM probes entirely. The assumption of normal and exponential background and signal distributions generally holds in practice, is justified from physical binding considerations, and the resulting background-subtracted signals preserve the overall shape and patterns of the unprocessed scatterplots without introducing additional low-intensity variability (Figure 2D). dChip uses a probeset-level probe-specific background model (MBEI), trained from all chips within a dataset, and defaults to using MM-subtracted PM intensities. As a result of the model-based approach, this method generates differing results depending on the populations of chips processed together. Although it is comparable to either MAS5 or RMA [7,8] in spike-in experiments, dChip does not support background subtraction prior to probe-level normalization and probeset summarization. Thus, RMA background subtraction was chosen for use in IRON, due to its non-specific binding signal deconvolution methodology, lack of introduced noise, and chip independence.

### Pair-wise iterative rank-order normalization minimizes introduced artifacts in biologically complex data

There are three major normalization methods that are commonly employed: linear scaling (MAS5), quantile normalization (RMA), and pair-wise rank-invariant normalization (dChip). Linear normalization is the simplest of the methods, which applies a global scaling factor to each chip (at the probeset level in MAS5) in order to scale all chips to the same trimmed mean intensity. Quantile normalization ranks the intensities for each chip, then replaces the intensities at each rank with the mean intensity for all probes of that rank across all chips, effecting a non-linear rank-dependent normalization. Pair-wise rank-invariant normalization normalizes all chips against a single reference chip by identifying a different subset of rank-invariant genes for each sample/reference chip pair, fitting a curve through the training set, then adjusting the intensities of the target chip in an intensity-dependent manner so that the fit curve will lie on the *sample* vs. *reference* diagonal of the scatterplot.

Linear normalization is unable to correct for non-linear, intensity-dependent differences in gene expression between chips, but can be applied to a single chip, independently of other chips. Quantile normalization assumes that differential gene expression is symmetric, in that there will be a roughly equal number of up and down regulated genes with equal magnitude distributions. Due to its population-based signal, it requires a moderately large number of chips in order to work well, and may introduce unexpected artifacts, particularly in outlier samples, in small experiments, or experiments in which different cell/tissue types are represented. Rank-invariant normalization makes similar assumptions to those of quantile normalization, since both are rank based, but can be applied to as few as two chips.

**Figure 3:**
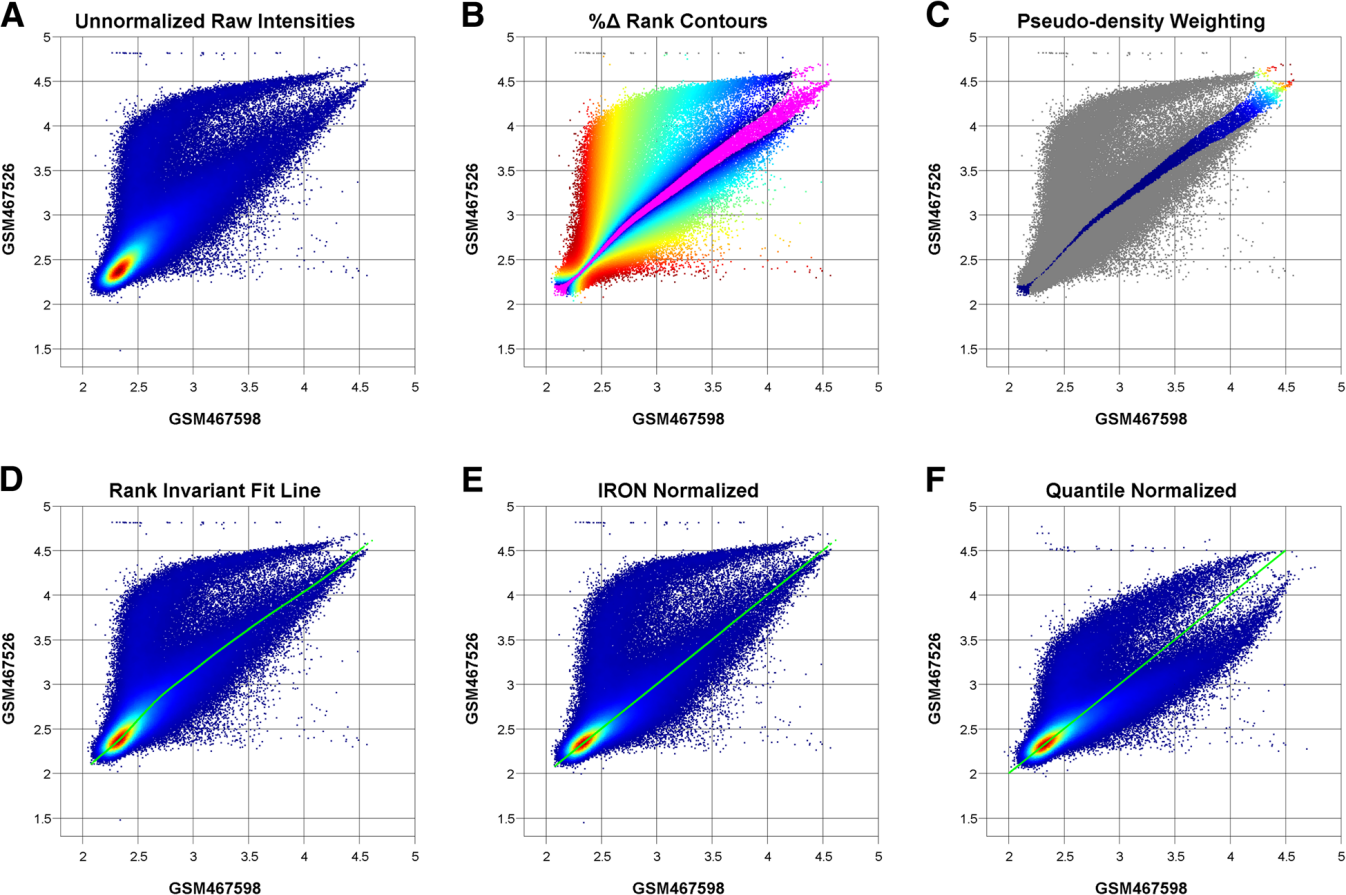
**IRON normalization.** Scatterplots of log_10_ non- background-subtracted probe intensities are used to demonstrate the IRON normalization algorithm. Points are colored by density (red: high, blue: low) in A, D, E, F. Initial points (**A**), are filtered in (**B**) to remove extreme low- and high- intensity points (grey). Iterative rank-order pruning (**B**) further removes outlier points at each iteration (red: high %Δ rank, blue: low %Δ rank), leaving the final training set (magenta) differing by ≤ 1 %Δ rank. Sparsely sampled regions (red) within the training set are up-weighted in (**C**), prior to fitting a smoothed piece-wise linear curve (green) in (**D**). Non-linear intensity-dependent scaling is applied to the sample (GSM467826) using the fit curve, so as to shift the fit curve onto the *X*,*Y* diagonal (**E**). The non-linear scaling resulting from quantile normalization (**F**), is unable to cope with the asymmetry between the samples, effectively fitting a line (diagonal in green) between the highest density distribution and the lesser density subpopulation, resulting in greater non-linear distortion than originally present in the unprocessed data.

Since linear normalization performs the least amount of manipulation to the original data, it is arguably the least destructive when its assumptions are violated. Quantile and rank-invariant normalization perform well when the symmetric distribution assumption holds, but can dramatically distort the data when this assumption is violated (Figure 3F). IRON normalization attempts to provide as flexible a solution as linear and traditional rank-invariant normalization, which are not limited to large homogenous datasets, while minimizing errors introduced from violations of the underlying assumptions of the algorithm.

Large numbers of genes changing in a single direction, and/or large magnitudes of change in one direction, displace the ranks of unchanged genes, causing the unchanged genes to exhibit large changes in rank. This is evident in Figure 3, where ~10% of the probes are highly up-regulated in sample GSM467526 of GEO [2] dataset GSE18864, creating an upper “arm”. Both traditional fixed-cutoff rank-invariant fitting (Additional file 1: Figure S1A), as well as quantile normalization (Figure 3F), effectively fit a line passing between the true high density diagonal and the secondary distribution arm. IRON iteratively decreases the rank difference cutoff, starting at the maximum observed difference in the dataset, until convergence to a set of probes that differ by ≤ 1% rank. This differs from previously described iterative rank-invariant methods, which iterate a fixed cutoff, or, in the case of dChip, a narrow range of strict cutoffs, until convergence. The iterative use of a gradually more stringent cutoff largely eliminates the problem of asymmetric changes inducing false-positive shifts in rank, since most of the offending outlier points are iteratively discarded before they can negatively impact the final rank-order analysis (Figure 3B). Interestingly, dChip, despite its average-rank dependent cutoff between 0.3%–0.7%, produced results that were more similar to IRON than to fixed-cutoff rank-invariant normalization (Additional file 1: Figure S1).

To observe the effects of background subtraction (specifically, RMA background subtraction) on probe-level normalization, we also examined normalization as it would occur within the IRON and RMA pipelines (Additional file 2: Figure S2). The same effects of violation of the symmetry assumption are observed in the background-subtracted figures as in the non- background-subtracted figure (Figure 3, Additional file 2: Figure S2). Since dChip subtracts background after probe-level normalization, and MAS5 normalizes at the probeset level, background-subtracted probe-level normalization comparisons are not applicable.

### MAS5 probeset summarization reduces both false-correlation and removal of biological signal

Probesets, often a collection of 10 or more PM/MM probe pairs, must be summarized into a single intensity value representative of the behavior of the set of probes as a whole, which reflects the expression of the target transcript. The most commonly used approaches are Tukey’s Biweight (MAS5) and Median Polish (RMA). Tukey’s Biweight is a weighted average of the individual log_2_ probe intensities, down-weighting probes more distant from the median of the probeset. This should be more tolerant of outlier probes/spots than an unweighted average [5]. RMA probeset summarization fits a linear additive model of *signal* + *probe-affinity* + *error* terms, using Median Polish to estimate the model parameters for each probeset across all chips. Giorgi *et al.*[9] have shown that RMA probeset summarization introduces false correlation, via the median polish procedure in high variability probesets yielding identical values across chips.

**Figure 4:**
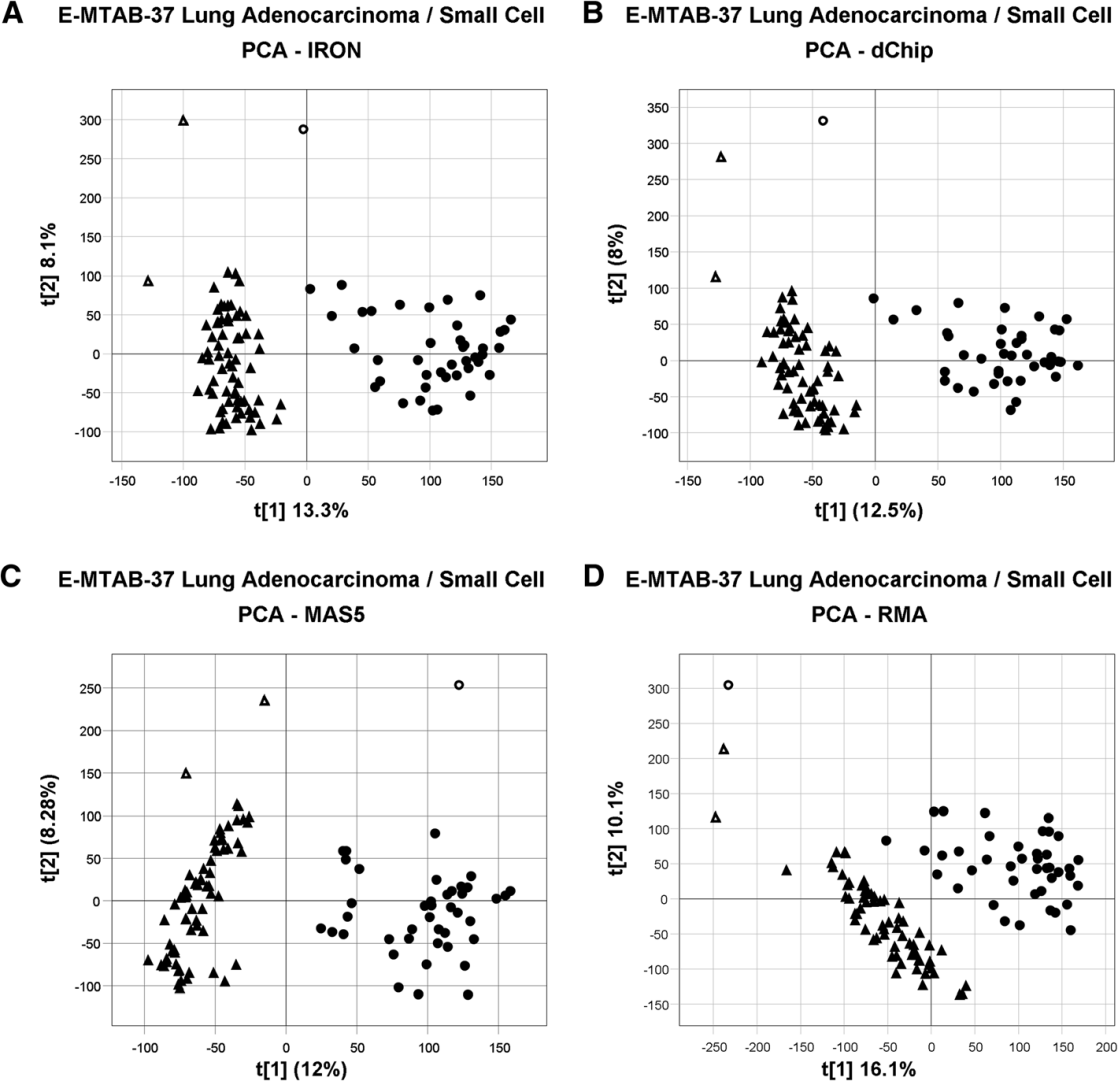
**Principle component analysis.** Lung adenocarcinoma derived samples (triangles) separate from small-cell derived samples (circles). Replicate outliers are designated by their respective unfilled shapes. (**A**) IRON normalized; adenocarcinoma samples cleanly separate from small-cell samples along the first principle component. Biological separation is captured almost entirely by the first principle component, with negligible separation along the second component. Outlier replicate samples group with their respective cohort. (**B**) dChip normalized; results are similar to those of IRON, but separation of the two groups is slightly less clean, and the small-cell outlier is on the adenocarcinoma side of the first principle component. (**C**) MAS5 normalized; adenocarcinoma and small-cell samples cleanly separate from one another, together with their respective outlier samples. Two distinct sub-clusters are evident in both the adenocarcinoma and small-cell derived samples, which are not observed in the results from other methods. (**D**) RMA normalized; adenocarcinoma and small-cell samples do not separate well along the first component, requiring a combination of both the first and second components to achieve separation. Replicate outlier samples group with themselves, rather than with their respective biological cohorts.

Additionally, we have observed that median polish often blurs the differences between biologically distinct groups of samples. This is exemplified in Figure 4, where we compare the results from a Principle Component Analysis (PCA) on the same dataset using four different post-processing pipelines: IRON, dChip, MAS5, and RMA. The separation between the two classes is completely in the first principle component for the IRON and MAS5 normalized data, while the separation of the two classes is spread between both components one and two for the dChip and RMA normalized data. dChip separates the samples similarly to IRON, suggesting that MBEI may not remove as much variation as median polish. Substituting median polish probeset summarization into the IRON pipeline produces a similar result to that seen for RMA (data not shown), indicating that median polish probeset summarization may degrade biological signal. This, together with the findings of Giorgi *et al.*[9], suggests that median polish may be overcorrecting by removing biologically-derived variation in addition to technically-derived variation. Given the undesirable behaviors of lack of chip-independence, introduction of false-correlation, and removal of biological signal, Tukey’s Biweight was chosen over median polish as the default IRON probeset summarization method.

### Probeset-level normalization corrects further intensity-dependent differences

Regardless of the background subtraction, probe-level normalization, and probeset summarization method used, we have observed that the resulting probeset intensities often exhibit similar patterns of non-linear intensity-dependent differences in signal levels as those of their underlying raw unprocessed data. Choe *et al*. [7] also commented on the high frequency of this occurrence, and demonstrated marked improvement in signal quality by applying an additional pass of pair-wise normalization at the probeset level. We observed similar positive effects, both visually and biologically (data not shown). Thus, IRON performs a final pass of pair-wise normalization at the probeset level, after all other processing has been performed.

### Comparison of techniques on spike-in benchmarks

The Affycomp III [10] and Golden Spike [7] spike-in benchmarks were used to compare the IRON pipeline to existing techniques. The Affycomp spike-ins are a small number of symmetrically altered transcript concentrations in a common background, with little normalization required between samples. The Golden Spike experiment, on the other hand, spikes in uni-directionally varying concentrations of 1309 out of 3860 transcripts (34%), resulting in significant violations of the symmetry assumption inherent to many normalization methods. As such, differences in Affycomp metrics may be driven by choice of background subtraction and probeset summarization methodology, while differences in the Golden Spike dataset results are driven by the ability of the normalization method to cope with violations of the symmetry assumption [11].

**Figure 5:**
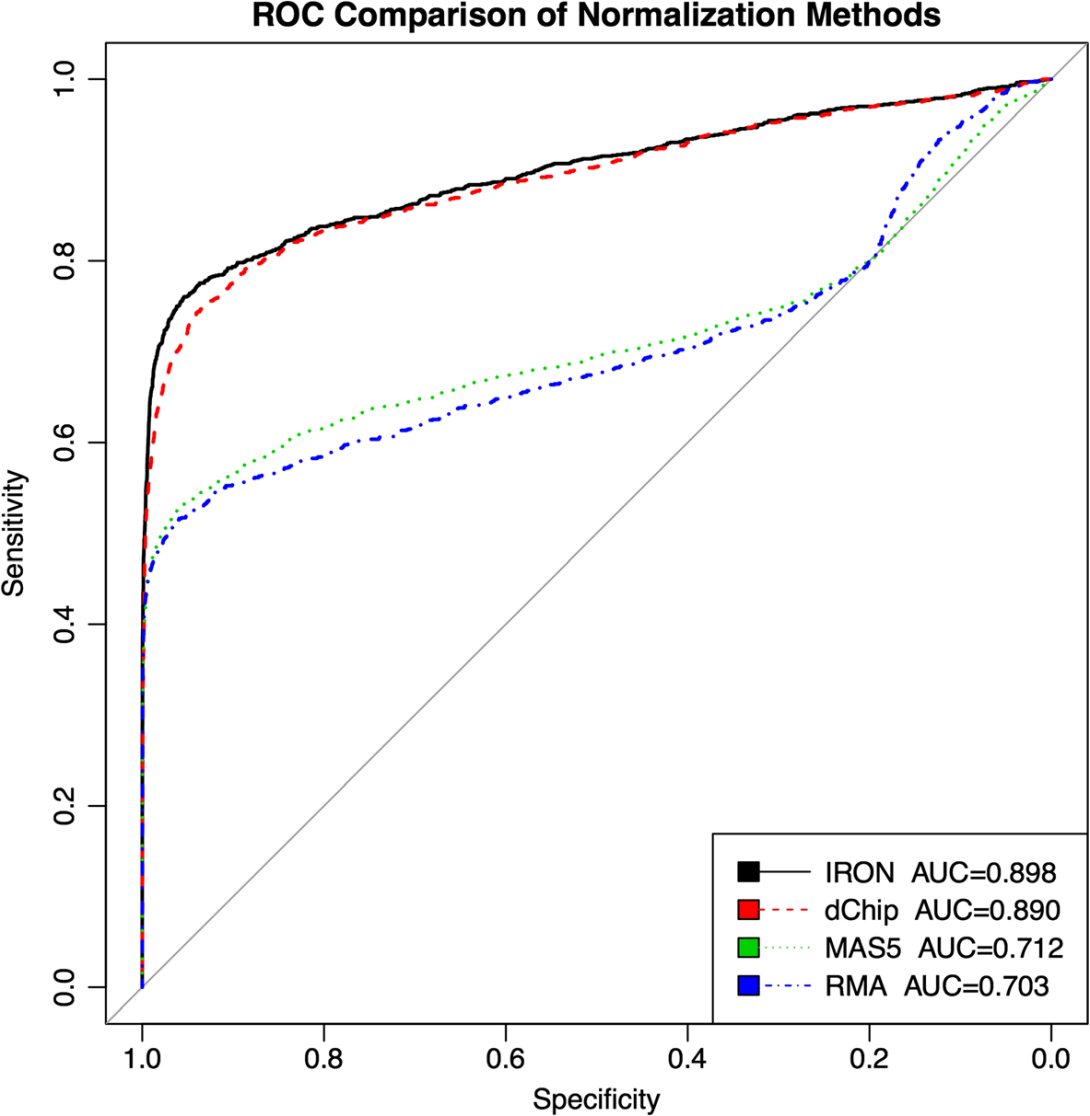
**Golden Spike experiment.** Post-processing pipelines involving pair-wise rank-invariant normalization methods (IRON: black, dChip: red) outperform methods that employ linear scaling (MAS5: green) or quantile normalization (RMA: blue).

Due to its combination of RMA background subtraction and Tukey’s Biweight probeset summarization, IRON performs somewhere between MAS5 and RMA in the Affycomp benchmark (Table 1). IRON is closer to RMA performance for metrics dominated by background subtraction (e.g. Median SD), and closer to MAS5 in metrics dominated by probeset summarization (e.g. AUC). The IRON approach thus performs similarly in Affycomp to MAS5, RMA, and dChip. AUC measurements of spike-in detection on the Golden Spike experiment (Figure 5) show IRON as the top-performer (AUC = 0.898), followed closely by dChip (0.890). Both IRON and dChip perform noticeably better than MAS5 (0.712) and RMA (0.703). Due to the asymmetric properties of the Golden Spike dataset, the performance of IRON and dChip can be attributed to the ability of their respective normalization procedures to accommodate asymmetric gene expression changes.

**Table 1:** Affycomp III results

		**HG-U95a**				**HG-U133a**			
**Metric #**	**Metric Name**	**MAS5-apt**	**IRON**	**RMA-apt**	**dChip**	**MAS5-apt**	**IRON**	**RMA-apt**	**dChip**
1	Median SD	0.59	0.20	0.11	0.32	0.29	0.14	0.07	0.20
2	null log-fc IQR	0.84	0.33	0.19	0.38	0.47	0.24	0.13	0.26
3	null log-fc 99.9%	4.46	1.59	0.57	10.83	4.01	1.42	0.40	9.61
4	Signal detect R^2^	0.86	0.82	0.80	0.61	0.91	0.90	0.90	0.65
5	Signal detect slope	0.71	0.67	0.63	0.91	0.77	0.69	0.68	0.98
6	low.slope	0.69	0.44	0.35	1.17	0.65	0.41	0.31	1.29
7	med.slope	0.81	0.82	0.76	0.87	0.71	0.68	0.71	0.75
8	high.slope	0.45	0.50	0.47	0.34	0.77	0.83	0.80	0.69
9	Obs-intended-fc slope	0.69	0.66	0.61	0.87	0.77	0.69	0.68	0.99
10	Obs-(low)int-fc slope	0.65	0.44	0.36	1.68	0.64	0.40	0.31	1.17
11	low AUC	0.00	0.03	0.40	0.04	0.00	0.08	0.51	0.08
12	med AUC	0.00	0.03	0.87	0.00	0.00	0.10	0.88	0.00
13	high AUC	0.00	0.00	0.46	0.00	0.00	0.13	0.93	0.00
14	weighted avg AUC	0.00	0.03	0.52	0.03	0.00	0.08	0.60	0.06

IRON generally performs in-between MAS5 and RMA, as expected, due to the mix of RMA background subtraction (RMA) and Tukey’s Biweight probeset summarization (MAS5). Metrics 1–10 are closer to RMA due to the use of RMA background subtraction, while metrics 11–14 are closer to MAS5 due to the use of Tukey’s Biweight probeset summarization.

Comparison of AUC measurements across the spike-in literature can be difficult, due to differences in how differentially expressed probesets are identified. These differences can lead to opposite and seemingly contradictory conclusions. As noted by Choe *et al*. [7], and also shown by Irizary *et al*. [12], MAS5 can perform significantly better or worse than RMA, depending on whether fold-change or variance-based metrics are used to determine differentially expressed (DE) probesets. Fold-change based methods are highly sensitive to variation within the low-intensity region, where small (noise level) changes in intensity can result in overly-large fold-changes. These probesets are identified as differentially expressed, which, in turn, results in low AUC measures. Thus, methods that minimize variation perform well in Affycomp, which uses only fold-change to determine DE probesets. Variance-based evaluation methods, such as those used in Choe *et al*. and Irizary *et al*. ([12], Figure 2D), as well as in this manuscript, do not implicitly favor minimization of low-intensity variation. Post-processing techniques in which variation is not as aggressively reduced could then potentially lead to increased sensitivity. Direct comparison of AUC results from different evaluation methods can be challenging, and we believe that evaluation must be performed in the larger context of how the various methods affect background-subtracted intensities, signal normalization, probeset summarized intensities, and other biological-signal related methods.

### Application

The simple, ideal spike-in experiments are far from capturing behavior observed in biologically complex data, particularly when it comes to heterogeneous samples such as human tumors. We have frequently observed difficulties with cancer datasets, particularly in publicly available data. The combination of tumor heterogeneity, batch effects, and differing protocols for generating the microarrays leads to less than ideal conditions for analysis. The choices in IRON for background subtraction, normalization, and probeset summarization were made on both theoretical grounds and empirical observations of behavior in existing datasets. Our goal is an algorithm that seeks to best preserve true differences between samples, including batch effects, while minimizing technical variation and processing-introduced artifacts. We have shown that the resulting combination of microarray normalization pipelines provides a robust method that is suitable for diverse datasets.

A challenge in expression normalization is the existence of large patient cohorts or cell line datasets that must be processed together. For instance, IGC [13] consists of ~2100 tumors, the ArrayExpress [3] cancer cell line dataset E-MTAB-37 has 950 samples, and our institution has collected over 19,600 GeneChips from tumors [14]. There are clearly diminishing returns from estimating model parameters (in methods such as RMA) from such large datasets [15]. The need for dataset-specific parameters/normalization is arguably necessary for specific tumor types. IRON addresses these issues by avoiding multi-chip calculations without sacrificing the advantages in precision from these approaches. The algorithm identifies a single median chip to normalize the set against. This is the only global analysis performed, and the remaining processing can be done serially or distributed across many parallel nodes.

Increased focus has been placed on avoiding methodological biases in analysis of gene expression. One area that is not typically highlighted is the normalization step. A validation of a gene expression signature should be completely independent of the process of generating the signature. However, in the case of RMA, building separate models of normalization for both training and test sets can lead to systematic differences due to the process. IRON avoids this difficulty through the use of a single reference sample. By incorporating this approach, any single sample can be successfully classified by normalizing against the median sample. While it is possible to take parameters from an existing RMA model [15,16] and apply them to new data, the drift in expression from the initial training set could negatively impact normalization of new samples. By normalizing against the median (reference) sample, this difficulty is minimized. We have observed little difference in gene expression estimates based on selection of the reference sample, so long as the chosen reference sample is not an outlying sample (data not shown).

The IRON algorithm is amenable to distributed processing. The selection of the median chip is disk and memory intensive, since it requires an all vs. all chip comparison which is difficult to efficiently parallelize. However, this is generally not a limiting step, as we have performed median chip analysis of ~15,700 chips (at the probeset level) on a single CPU core, using 5.7 GB of memory in less than 6 hours. If, in the future, data size scales more rapidly than memory capacity, the memory limitation could be easily addressed through techniques such as sparse sampling of probes and probesets with minimal impact on the accuracy of median chip selection. The normalization itself can be run in a highly parallel fashion in which every chip is processed independently. Each pair-wise normalization does require an iterative procedure that must converge. However, both in computational time and memory requirements, the maximum amount needed scales linearly with the number of samples analyzed, normalizing greater than four Affymetrix HG-U133 Plus 2.0 chips per minute, per core, on modern hardware.

## Conclusions

Each of several commonly used microarray normalization pipelines (MAS5, RMA, dChip), contain background subtraction, normalization, and probeset summarization algorithms that are more or less desirable compared to others. The new pipeline presented here, IRON, recombines these algorithms, extending the pair-wise normalization procedure with a new iterative rank-order method, so as to limit the amount of potential harm to the processed data while maintaining the ability to correct for common technical artifacts. The intensities of each chip, while still dependent on the choice of reference chip, are independent from those of other chips, allowing for processing of small numbers of samples (≥ 2), and avoiding the problem of outlier chips negatively impacting the quality of other chips. IRON should be generally applicable to any dataset, whether it contains large or small numbers of samples, and whether it contains highly similar or dissimilar samples.

IRON is implemented as part of the libaffy C library and set of tools [17]. Source code for these tools, along with pre-compiled binaries for selected platforms, is available at [http://gene.moffitt.org/libaffy/] under the GNU Public License (GPL).

## Methods

### Overview of processing pipeline

The IRON array processing pipeline employs RMA background subtraction [4], Tukey’s Biweight probeset summarization [5], and a novel pair-wise iterative rank-order normalization (IRON) method that is able to largely handle violations of the symmetry assumption implicit in quantile normalization and traditional pair-wise normalization algorithms. For each chip, IRON normalization performs a pair-wise (*sample* vs. *reference*) normalization against a common reference array (Figure 3). For each pair-wise normalization, a smoothed piece-wise linear fit is performed against a core set of non-differentially expressed probes (Figure 3D), identified through an iterative rank-order procedure (Figure 3B). The fit line is then used to non-linearly scale the sample intensities (Figure 3E). Normalization is applied at both the probe and probeset level. The major improvement of IRON over previous rank-based algorithms is its iterative rank-order refinement of the training set. The common reference array is selected by performing an all vs. all chip comparison to identify the median chip.

### Reference chip selection

A reference chip is selected by first calculating all vs. all root mean squared distances (RMSD) between chips, with each chip consisting of a vector of all raw log_2_ probe intensities, excluding undefined and quality control probes. Tukey’s Biweight probeset summarization can, optionally, be performed prior to RMSD calculations to greatly reduce memory usage in large datasets. For each chip, the RMSD from all other chips is calculated using the previous pair-wise RMSDs. The chip with the smallest RMSD is chosen as the median chip and used as the reference chip during normalization. Use of pair-wise RMSD performs better for the selection of a median chip than pair-wise correlation-based distance metrics, due to its ability to select a chip of median brightness while concurrently selecting for minimal relative curvature (data not shown).

### Pair-wise normalization

Each chip is then processed independently. First, RMA background subtraction is performed. Then, the set of probes to be used for training the best-fit non-linear curve is identified. Probes that are not part of a probeset are excluded, as are masked probes. Of the remaining probes, probes with the lowest intensity value are excluded, as well as probes with the highest intensity value and any probes deemed to be saturated (intensity > 64,000 for 16-bit data). Iterative rank-order pruning is then performed vs. the reference chip, removing the most highly rank-divergent probes (max percentile difference per iteration, minus 0.5%), to a convergence of 1% rank-invariance.

The remaining training points are then sorted by log(X*Y), where *X* is the intensity on the reference chip and *Y* the intensity on the sample chip. A sliding window 10% the size of the training set is then used to calculate a series of best-fit *log(X/Y)* vs. log(X*Y) weighted least-squared lines. As observed by Li and Wong [6], sparsely sampled regions, particularly at high intensities, require up-weighting in order to achieve better fits in these regions, since otherwise the fits would be dominated by the higher density areas of the scatterplot. To adjust for density, points are weighted by *σ*_*avg*_^4^ prior to fitting, where *σ*_*avg*_ is the average standard deviation calculated from each sliding log(X*Y) window (1% of training set) containing the point. Although *σ*_*avg*_^4^ performs well for all microarray data we have encountered, user-definable smaller exponents may be more appropriate for small training sets (hundreds of points) of more homogenous density. For each point in the training set, the slope and offset of all best-fit lines containing the point are averaged and applied to the log(X*Y) value to yield the fit *log(X/Y)* correction factor for that point. This produces a similar result to the commonly used LOESS [18] fit, but results in better fit lines for the purpose of normalization (data not shown).

The distance from the fit curve, *d*, is calculated for each point as (*log(X/Y)*_*fit*_*– log(X/Y)*_*obs*_), where *log(X/Y)*_*fit*_ and *log(X/Y)*_*obs*_ are the fit and original observed correction factors. The final set of adjusted (*log(X), log(Y)*) points describing the non-linear fit curve, approximating the shortest path of highest density through the scatterplot, is generated by projecting the observed training probe coordinates (*log(X*_*obs*_*), log(Y*_*obs*_*)*) onto the best-fit curve by (*log(X*_*proj*_*) = log(X*_*obs*_*) + ½ d*, *log(Y*_*proj*_*) = log(Y*_*obs*_*) – ½ d*). Where multiple probes project onto the same fit *Y* coordinate, their fit correction factors are averaged. Every probe on the chip is then normalized by applying the final correction factor corresponding to the nearest point (nearest in *Y*) on the fit curve, using linear interpolation between fit points. For points outside the bounds of the training set, the averaged correction factor for the first 10 or last 10 fit points is used for points below or above the training set, respectively.

### Querying method popularity within public data repositories

The relative popularity of normalization methods was assessed by querying the GEO [2] and ArrayExpress [3] websites on June 28th, 2012 for the following keywords: RMA, MAS5, dChip, GCRMA, PLIER, VSN. ArrayExpress queries were limited to RNA assays. Both sets of queries resulted in the following top-four ordering: RMA > MAS5 > dChip > GCRMA.

Figure 1 was created in Microsoft PowerPoint 2003 (Microsoft Corporation, Redmond, WA, USA). All results pertaining to MAS5 and RMA were derived from data processed with the Affymetrix Power Tools software, v1.12.0 (Affymetrix, Inc., Santa Clara, CA, USA). All results pertaining to dChip were derived from data processed with the December 17th, 2010 Windows binary. All plots and PCA analyses were generated with Evince, v2.5.5 (UmBio AB, Umeå, Sweden). Final figures were composited using Inkscape, v0.48 (http://inkscape.org/).

The data for Figures 2, 3, Additional file 1: Figure S1, and Additional file 2: Figure S2 were generated using GEO [2] dataset GSE18864, after excluding GSM467575 as a bad chip. Both IRON and non- background-subtracted quantile normalization were performed using libaffy. IRON normalization was performed versus chip GSM467598. dChip normalization was performed against GSM467598 for Figure 3, Additional file 1: Figure S1, and Additional file 2: Figure S2, and against the default median brightness chip for all other analyses. The data for Figure 4 was generated from ArrayExpress [3] dataset E-MTAB-37, using the subset of adenocarcinoma and small-cell lung tumor-derived cell line chips. IRON normalization was performed versus sample NCI-H1437-Rep3. Three samples (NCI-H1355-Rep1, NCI-H1792-Rep2, NCI-H2107-Rep1), denoted with ‘X’ symbols in the figure, were outlier technical replicates. Three technical replicates were run for most cell lines, and these three samples were unlike the other two replicates for their respective cell lines. These outlier replicates were left in the analysis to highlight the effect of choice of post-processing algorithm on the behavior of the principle component analysis. Removal of the outliers prior to PCA analysis does not noticeably impact the behavior of the non-outliers (data not shown).

Normalized expression data for Figure 5 was loaded into R 2.15.1. The GoldenSpike package (v0.5) was used and modified to evaluate spike-in probesets. Briefly, cyberT was used to identify differentially expressed probesets, and the statistic was used as the score for ROC analysis. ROC and AUC analysis was performed using the pROC package [19], using spike-in probesets as positives (cases), background / not spiked-in probesets as negatives (controls), requiring cases to have larger scores than controls.

Table 1 was generated by submitting MAS5, RMA, and IRON results to the Affycomp III web-server [10,20], then entering the results into Microsoft Excel 2007 (Microsoft Corporation, Redmond, WA, USA). dChip results were taken directly from the Affycomp III competition results webpage.

## Abbreviations

IRON: Iterative Rank-Order Normalization; MM: Mis-Match probe; PM: Perfect Match probe; PCA: Principle Component Analysis; GPL: GNU Public License; RMSD: Root Mean Squared Deviation

## Competing interests

The authors declare that they have no competing interests.

## Authors’ contributions

EAW developed and implemented algorithms, performed analysis and interpretation of data, and wrote the manuscript. SAE consulted on software implementation. SAE and AEB consulted on interpretation of data and drafting of the manuscript. AEB and DAF contributed to drafting of the manuscript and final approval. SAE and DAF contributed to acquisition of microarray data and funding. All authors read and approved the final manuscript.

## Supplementary Material

Additional file 1: Figure S1 Background-subtracted normalization. Scatterplots are of log_10_ background-subtracted probe intensities. Points are colored by density (red: high, blue: low). Background subtraction was performed prior to normalization, reflecting the behavior of normalization within the IRON (**A**) and RMA (**B**) pipelines. IRON normalization centers the highest density distribution along the diagonal (thick green line), while quantile normalization centers the region between the two density distributions along the diagonal. Although generally down-shifted in intensity, the same patterns are observed in the background-subtracted data as in non- background-subtracted examples. Click here for file

Additional file 2: Figure S2 IRON vs. fixed-rank pair-wise normalization. Scatterplots are of log_10_ non- background-subtracted probe intensities. Points are colored by density (red: high, blue: low). Iterative rank order normalization (**B**), with a gradually decreasing rank-difference cutoff, is more robust to symmetry violations than a fixed rank-difference cutoff of 0.5% (**A**), and better centers the distribution of highest density along the diagonal (thick green line) than dChip (**C**). Click here for file
