# Supplementary figures and images for: Iterative rank-order normalization of gene expression microarray data
Source: BMC Bioinformatics. 2013 May 7;14:153. doi: 10.1186/1471-2105-14-153 (PMC3651355; doi:10.1186/1471-2105-14-153)

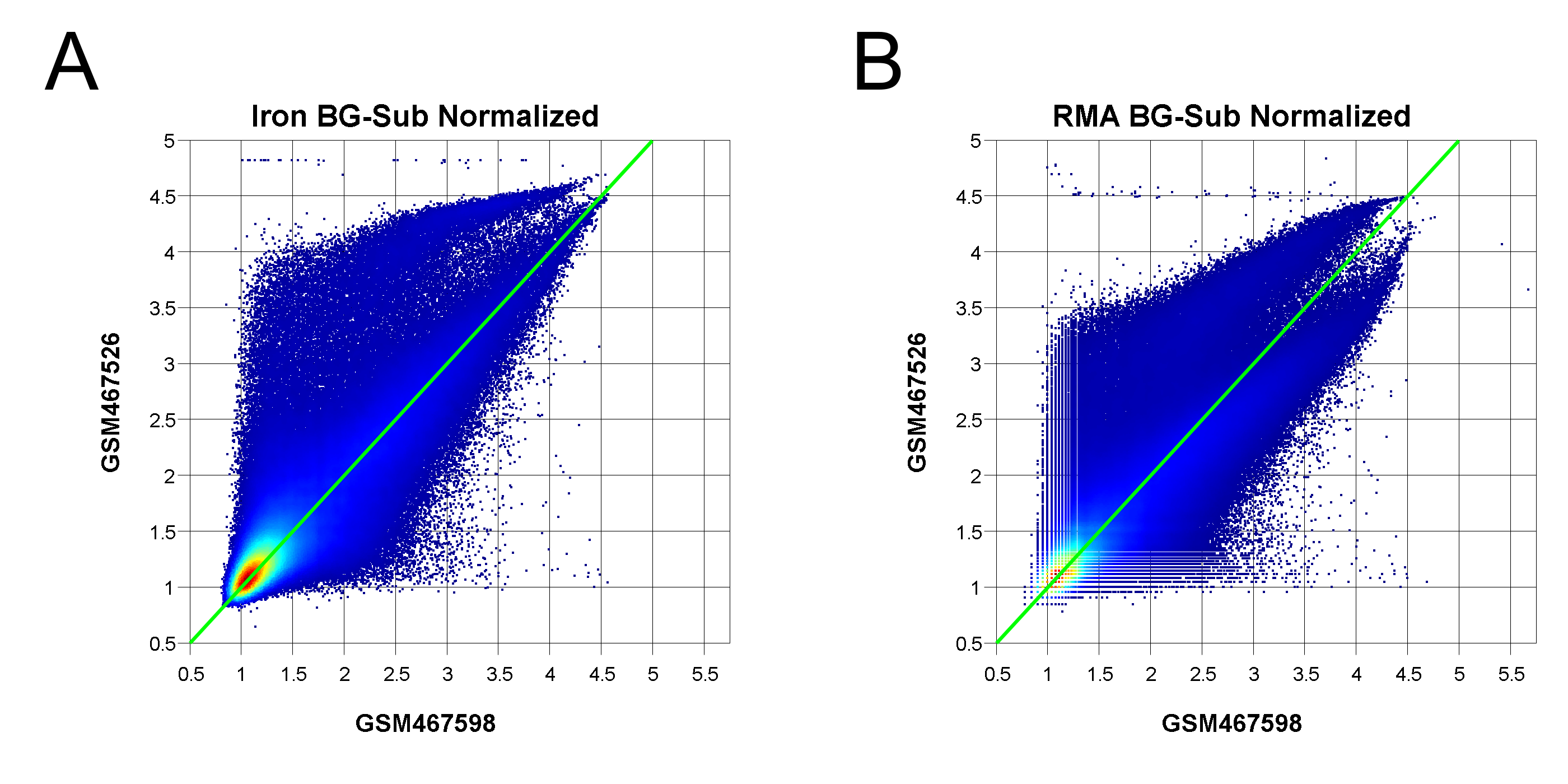

Supplement: Additional file 1: Figure S1 — Background-subtracted normalization. Scatterplots are of log10 background-subtracted probe intensities. Points are colored by density (red: high, blue: low). Background subtraction was performed prior to normalization, reflecting the behavior of normalization within the IRON (A) and RMA (B) pipelines. IRON normalization centers the highest density distribution along the diagonal (thick green line), while quantile normalization centers the region between the two density distributions along the diagonal. Although generally down-shifted in intensity, the same patterns are observed in the background-subtracted data as in non- background-subtracted examples. [file 1471-2105-14-153-S1.png]

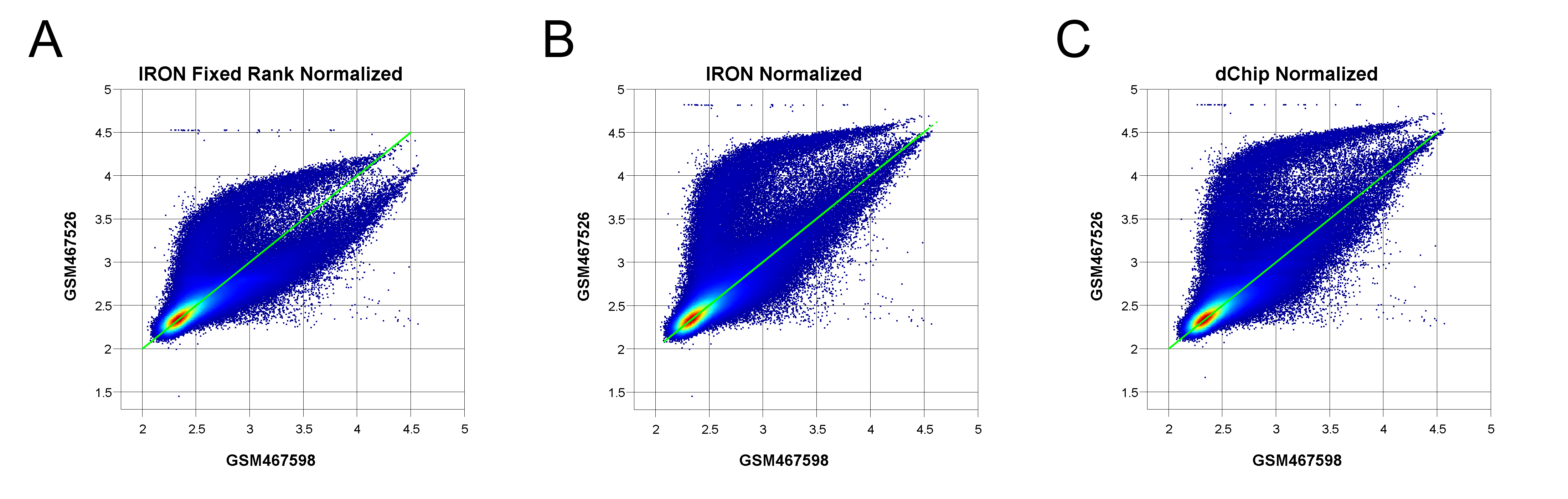

Supplement: Additional file 2: Figure S2 — IRON vs. fixed-rank pair-wise normalization. Scatterplots are of log10 non- background-subtracted probe intensities. Points are colored by density (red: high, blue: low). Iterative rank order normalization (B), with a gradually decreasing rank-difference cutoff, is more robust to symmetry violations than a fixed rank-difference cutoff of 0.5% (A), and better centers the distribution of highest density along the diagonal (thick green line) than dChip (C). [file 1471-2105-14-153-S2.png]
